# MRI-based risk stratification for predicting overall survival in pancreatic ductal adenocarcinoma

**DOI:** 10.1186/s13244-025-02088-1

**Published:** 2025-09-24

**Authors:** Haitao Sun, Jianbo Li, Lei Li, Peng Wang, Qiying Tang, Tianyu Lu, Jun Han, Zongyu Xie, Yiheng Zhou, Kai Liu, Mengsu Zeng, Minping Hong, Yaolin Xu, Jianjun Zhou

**Affiliations:** 1 Department of Radiology, Zhongshan Hospital, Fudan University, Shanghai Institute of Medical Imaging, No. 180 Fenglin Road, Xuhui District, Shanghai, 200032 China; 2 Department of Radiology, Zhongshan Hospital (Xiamen), Fudan University, Xiamen Municipal Clinical Research Center for Medical Imaging, Fujian Province Key Clinical Specialty for Medical Imaging, Xiamen Key Laboratory of Clinical Transformation of Imaging Big Data and Artificial Intelligence, Xiamen, 361015 China; 3 Department of Radiology, Fengyang County People’s Hospital, Chuzhou, China; 4 Department of Radiology, Affiliated Hospital of Jiangnan University, Wuxi, 214122 China; 5 Department of Radiology, The First Hospital of Jiaxing, Affiliated Hospital of Jiaxing University, Jiaxing, China; 6 Department of Radiology, The First Affiliated Hospital of Bengbu Medical University, Bengbu, China; 7 Wuxi School of Medicine, Jiangnan University, Wuxi, 214122 China; 8 Department of Radiology, Jiaxing Hospital of Traditional Chinese Medicine Affiliated to Zhejiang Chinese Medical University, Jiaxing, China; 9 Department of pancreatic surgery, Zhongshan Hospital, Fudan University, No. 180 Fenglin Road, Xuhui District, Shanghai, 200032 China

**Keywords:** Pancreatic ductal adenocarcinoma, Magnetic resonance imaging, Resectable, Prognosis, Model

## Abstract

**Purpose:**

To develop and validate a magnetic resonance imaging (MRI)-based pancreatic risk stratification model (M-PRiSM) for prognosis prediction and therapy guidance in pancreatic ductal adenocarcinoma (PDAC).

**Materials and methods:**

In this retrospective multicenter study, a total of 540 patients with PDAC were enrolled. A Cox proportional hazards model was used to identify significant clinical-radiological predictors and develop an M-PRiSM risk score system. The performance of the model was assessed using Harrell’s C-index, time-dependent area under the curve (AUC), and calibration curves, and was compared with existing clinical prediction metrics, including the 8^th^ American Joint Committee on Cancer (AJCC) staging and CA19-9 levels.

**Results:**

540 PDAC patients were split into a development cohort (*n* = 282) and an internal validation cohort (*n* = 122), with 136 patients forming external cohort A (*n* = 80) and B (*n* = 56). The model, incorporating CA19-9 (hazard ratio (HR), 1.604, *p* = 0.047), margin (HR, 1.918, *p* = 0.001), tumor size (HR, 1.308, *p* = 0.003), and venous enhancement ratio (VER) (HR, 0.605, *p* = 0.047), was developed, showing superior predictive accuracy for OS with C-indexes of 0.73, 0.70, and 0.70, and 0.68 across four cohorts. The model outperformed the 8th AJCC staging and CA19-9 alone across validation cohorts (*p* < 0.001). High-risk patients exhibit the worst overall survival (OS) and a higher frequency of adverse pathological features, but benefited significantly from adjuvant therapy, with improved survival outcomes.

**Conclusion:**

The M-PRiSM model effectively predicts postoperative OS and identifies PDAC patients likely to benefit from adjuvant treatment.

**Critical relevance statement:**

The M-PRiSM model, utilizing MRI and clinical features, enables preoperative risk stratification for overall survival in pancreatic ductal adenocarcinoma, offering a generalizable tool to guide personalized adjuvant therapy and enhance prognostic assessment.

**Key Points:**

- The M-PRiSM model can effectively predict pancreatic ductal adenocarcinoma survival.
- The M-PRiSM model showed strong generalizability in external validations.
- The M-PRiSM model stratifies patients for guiding adjuvant therapy for improved outcomes.

**Graphical Abstract:**

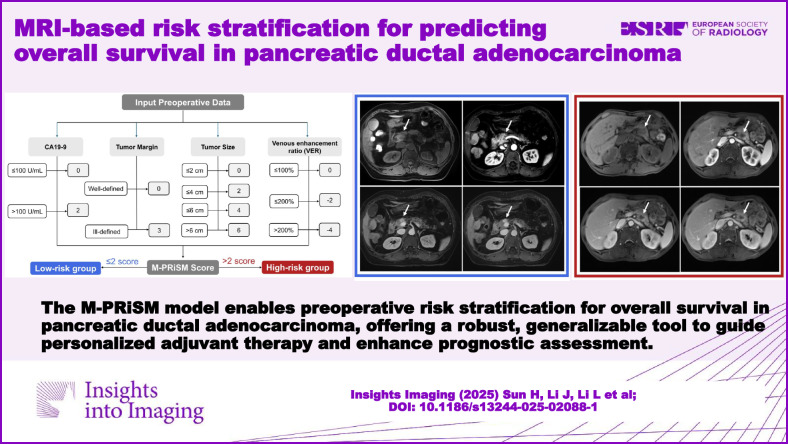

## Introduction

Pancreatic ductal adenocarcinoma (PDAC) is a major oncology challenge, with a 5-year overall survival (OS) rate of ~10–12% [1]. Despite surgical resection and chemotherapy improving outcomes, long-term recurrence and mortality remain high [2]. Accurate preoperative risk stratification is critical for surgical candidates to assess postoperative complications, recurrence-free survival (RFS), and OS, guiding multidisciplinary treatment strategies [3, 4].

Against the backdrop of rapid artificial intelligence advancement, CT or MRI-based radiomics and deep learning models have increasingly been employed to predict various aspects of PDAC aggressiveness, such as pathological grading, Ki-67 expression status, and genetic phenotype, as well as lymph node metastasis and overall prognosis, including RFS and OS [5–7]. Despite their promise, these models encounter problems such as the need for extensive manual image annotation, high computational demands, and limited interpretability and generalizability, which currently restrict their broader clinical practice.

Noninvasive imaging, particularly contrast-enhanced CT and MRI, offers valuable insights into the biological behavior and prognosis of PDAC [8, 9]. For instance, morphological and enhancement features in preoperative contrast-enhanced CT, including tumor size, adjacent organ invasion, regional lymph node metastasis, and ring-like enhancement, are associated with recurrence and poor outcomes after curative resection [10]. Conversely, contrast-enhanced MRI can more comprehensively reflect the intrinsic biological information of tumor cells, which may be more conducive to assessing the aggressiveness and prognosis of the tumor. For instance, MRI-detected necrosis has been closely associated with aggressive tumor differentiation and higher tumor cellularity in PDAC [11]. Additionally, features such as rim enhancement and the presence of intratumoral fluid-containing areas have been correlated with RFS or OS rates in PDAC patients post-resection [12]. Emerging evidence supports the potential of radiological-clinical models for prognostic risk stratification. A prior CT-based risk scoring system integrating clinical and imaging variables achieved a C-index of 0.68 for predicting RFS, surpassing traditional pathologic grading [13]. However, there are currently no large-sample, multicenter studies on clinical-radiological models based on MRI features to predict long-term OS in PDAC patients.

Hence, this study aims to develop and validate an MRI-based pancreatic risk stratification model (M-PRiSM) to predict long-term OS in retrospective, multicenter cohorts. By integrating accessible MRI features with clinical parameters, we seek to provide an interpretable, clinically translatable tool to guide personalized treatment decisions for PDAC patients.

## Materials and methods

The retrospective study conducted at Zhongshan Hospital, Fudan University, received approval from the institutional review board (B2024-250R) and was exempt from the necessity of obtaining written informed consent. Our investigation adhered to the TRIPOD (transparent reporting of a multivariable prediction model for individual prognosis or diagnosis) guidelines.

### Patients

**Fig. 1:**
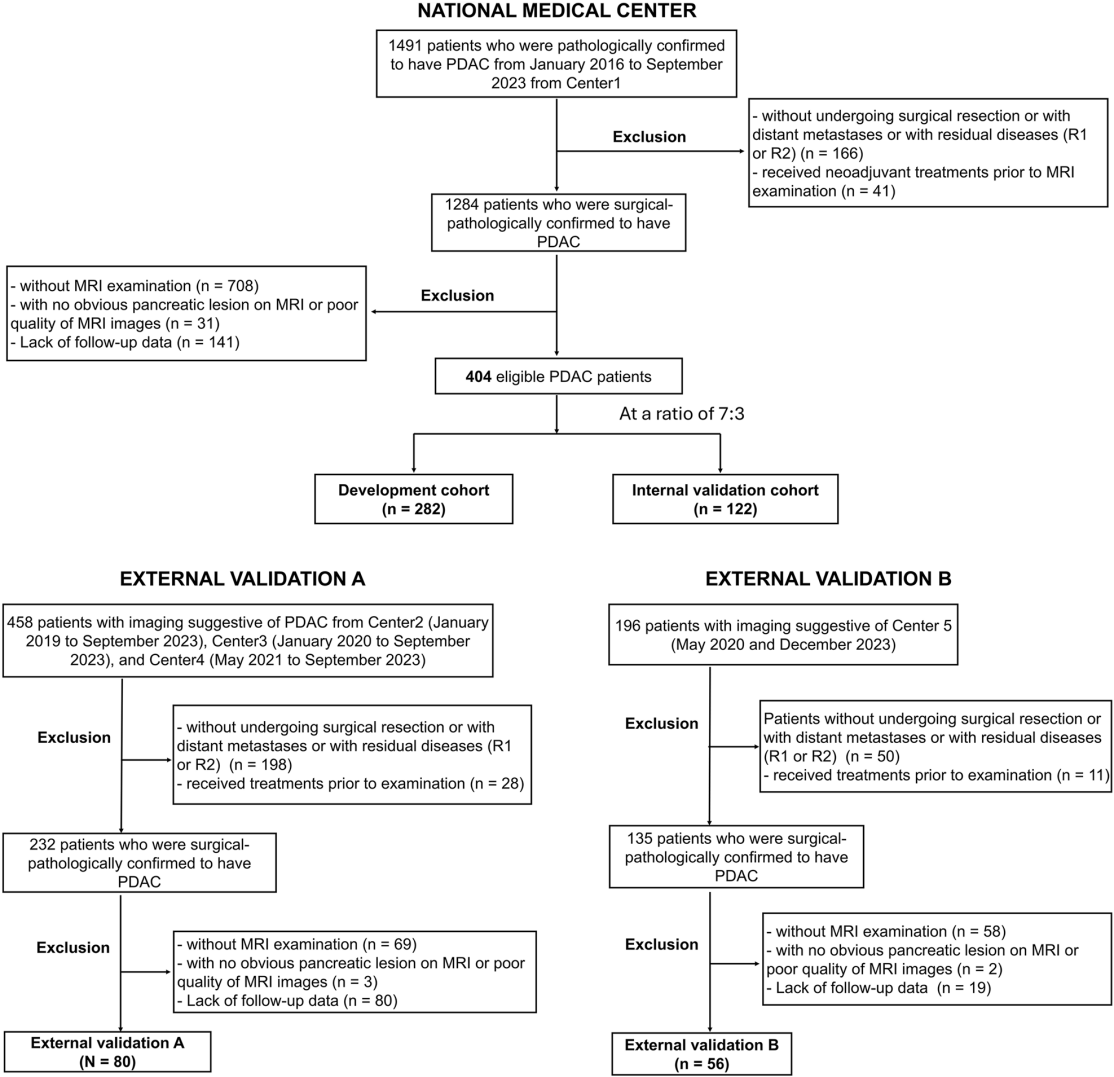
Flowchart of the study population

Data were collected from 1491 patients with pathologically confirmed PDAC at Zhongshan Hospital, Fudan University (center 1) from January 2016 to September 2023. Additionally, 654 PDAC patients were reviewed from four other centers formed two external validation cohorts: External validation A included patients from First Affiliated Hospital of Bengbu Medical University (center 2, January 2019–February 2024), Affiliated Hospital of Jiangnan University (center 3, January 2021–January 2024), and The First Hospital of Jiaxing (center 4, January 2020–September 2023); External validation B included patients from Xiamen Branch of Zhongshan Hospital (center 5, May 2020–December 2023). Inclusion criteria were pathologically confirmed PDAC via surgical resection, preoperative contrast-enhanced MRI within 2 months before surgery, and complete clinicopathological and prognostic data. Exclusion criteria included preoperative treatment, no surgical resection or R1/R2 resection, missing or poor-quality MRI, survival follow-up less than 90 days, or missing prognostic data. Details are in Fig. 1.

### MRI acquisition

The MRI scans were obtained using different 1.5- or 3.0-T MRI systems owing to the long-term and multicenter study design. Contrast-enhanced sequences were acquired following an intravenous injection of a gadolinium-based agent (e.g., Gd-DTPA or gadobenate dimeglumine) at 0.1 mmol/kg (1–2 mL/s). Appendix S1, Table E1, and E2 provide detailed information on the MRI protocols.

### Clinicopathological variables

The present study examined a range of demographic, pathological, and laboratory parameters. Demographic and laboratory parameters encompassed age, sex, body mass index (BMI), diabetes, hypertension, and serum concentrations of cancer antigen 19-9 (CA19-9), total bilirubin (TBil), and albumin levels.

Pathological parameters comprised the eighth edition of the American Joint Committee on Cancer (AJCC) staging, AJCC 8th pT stage, AJCC 8th pN stage, histologic grade, pathological vascular invasion, peripheral nerve infiltration, and pathological fatty infiltration (PFI).

### Image analysis

Two abdominal radiologists (with 10 and 19 years of experience in pancreatic MRI, respectively) retrospectively reviewed the images. The reviewers were aware that the patients had primary pancreatic cancer, but were unaware of all clinical, laboratory, and pathological information. The two reviewers independently assessed various imaging characteristics of each PDAC. When discrepancies arose between their evaluations, a third senior radiologist (with 30 years of experience in pancreatic MRI) re-evaluated the images, leading to a final consensus.

The MRI features of tumors were evaluated as follows: (a) location (head/body or tail); (b) margin (well-defined/ill-defined); (c) tumor size; (d) rim enhancement (e) enlarged lymph nodes at imaging, (f) main pancreatic duct (MPD) dilation, (g) intrahepatic bile duct (IBD) dilation, (h) pancreatic atrophy, (i) peripancreatic fat infiltration, (j) superior mesenteric vein (SMV)/portal vein (PV) contact (k) cystic degeneration, (l) hemorrhage, (m) necrosis. Detailed explanations for the imaging features above are presented in Appendix S2. Tumor signal intensity (high, iso, or low) was evaluated across T1/T2-weighted, arterial, venous, delayed, and diffusion-weighted images, compared to normal pancreatic parenchyma. The arterial enhancement ratio (AER) was calculated as (SI_arterial_ − SI_pre_) /(SI_pre_), and the venous enhancement ratio (VER) and delayed enhancement ratio (DER) were calculated as (SI_venous_ − SI_pre_)/(SI_pre_) and (SI_delayed_ − SI_pre_) /(SI_pre_), respectively. SI_pre_, SI_arterial_, SI_venous_, and SI_delayed_ represent the signal intensities (SI) in the pre-contrast, arterial, venous, and delayed phases, respectively.

The two radiologists measured apparent diffusion coefficient (ADC) and signal intensities (SI_pre_, SI_arterial_, SI_venous_, and SI_delayed_) by manually outlining regions of interest (ROIs) at the tumor’s maximum diameter, avoiding necrosis, cystic changes, or hemorrhage. ROIs covered the largest tumor area on two consecutive slices, with measurements averaged across slices and reviewers for final values [14, 15]. The final values were recorded as the means of two reviewers for further study.

### Patients outcomes

OS was the time from surgery to death or last follow-up, and RFS was from surgery to first recurrence or metastasis. Patients had clinical evaluations, serum CA19-9 tests, and contrast-enhanced CT/MRI every 3 months in the first postoperative year, then every 3–6 months, with a minimum 3-month follow-up. To reduce selection bias, patients alive without relapse or death by June 30, 2024, were censored.

### Statistical analysis

PDAC patients from center 1 were randomized into development and internal validation cohorts (7:3 ratio), while patients from four other centers formed two external validation cohorts. The 7:3 split was used to ensure sufficient data for model development while retaining a subset for internal validation. All eligible patients within the study period were included, eliminating the need for sample size calculation. Missing data (< 10%, mainly pathological features) were handled using multiple imputations with Markov chain Monte Carlo methods.

Continuous variables were dichotomized by normal ranges and reported as means ± SD (normal) or medians with IQR (non-normal). Categorical variables were proportions. Group comparisons used *t*-test or Mann–Whitney *U* for continuous variables and *χ*² for categorical variables. Inter-reader agreement used Cohen’s kappa (categorical) and intraclass correlation (continuous, bidirectional mixed model; Appendix S3.1).

We initially performed univariate Cox proportional hazards regression analyses to identify predictor variables significantly associated with the outcome. Variables that demonstrated statistical significance in these analyses were then included in a multivariate Cox proportional hazards model. The detailed process is shown in Appendix S3.2. Following the assessment of collinearity among the variables, the variable VER was retained and incorporated into the final multivariate Cox regression analysis (Fig. E1). The final multivariate model was fitted using the predictors selected through this process [16]. A risk scoring system was developed based on the multivariate Cox proportional hazards model, using the smallest coefficient as a reference and rounding scores down to the nearest integer [13, 17]. To simplify calculations, the regression model was approximated as a linear model, from which coefficients were extracted. A tumor size ≤ 2 was assigned a risk score of 0, with other groups proportionally adjusted relative to this baseline using their respective regression coefficients based on objective clinical practice [13]. Each variable was assigned a point value proportional to its estimated hazard ratio, ensuring that the scoring system closely reflects the relative contribution of each risk factor to OS. Using M-PRiSM medians allows participants to be stratified into high-risk and low-risk groups.

Model performance was assessed by examining both discrimination and calibration. Discrimination was evaluated using receiver operating characteristic (ROC) curves, with performance quantified by Harrell’s concordance index (C-index), the area under the time-dependent ROC curve (AUC), and decision curve analysis (DCA). The detailed analysis procedure was shown in Appendix S3.3.

Patients were divided into high- and low-risk groups using the M-PRiSM risk score. Subgroups within these groups were analyzed for long-term survival based on adjuvant treatment status. Kaplan–Meier survival curves were generated for each group and subgroup across datasets, with differences assessed via the log-rank test.

All statistical evaluations were performed using R software (version 4.4.3) and GraphPad Prism 8. A two-sided *p*-value of less than 0.05 was deemed statistically significant.

## Results

### Patient baseline characteristics

Table 1 summarizes demographics and baseline clinical characteristics of 402 eligible PDAC patients from 1491 screened, divided into development (*n* = 282; median age 66 (IQR 59–71); 158 men) and internal validation (*n* = 122; median age 66 (IQR 59–71); 77 men) cohorts at a 7:3 ratio. Additionally, 136 patients were enrolled in external validation cohorts: A (*n* = 81; median age 63 (IQR 59–69.2); 36 men) and B (*n* = 56; median age 66 (IQR 58.8–72); 23 men). Median follow-up for OS and RFS was: development (505 days (IQR 255–933), 359 days (IQR 168–720)), internal validation (543 days (IQR 316–1065), 349 days (IQR 181–832)), external A (384.5 days (IQR 178.5–611), 492.5 days (IQR 213–655)), and external B (479 days (IQR 223–711.5), 684.5 days (IQR 431–838.5)). Additional clinicopathologic and radiological data are in Tables 1 and E3.

**Table 1:** Baseline clinicopathological characteristics

Parameters	Development (*n* = 282)	Internal validation (*n* = 122)	*p*-value	External validation A (*n* = 80)	External validation B (*n* = 56)
Age (years)^a^	66 (59, 71)	66 (59, 72)	0.369	63 (59, 69.2)	66 (58.8, 72)
Sex			0.120		
Female	124 (44.0)	45 (36.8)		44 (55.0%)	33 (58.9%)
Male	158 (56.0)	77 (63.2)		36 (45.0%)	23 (41.1%)
BMI (kg/m²)^a^	22.35 (20.7, 24.4)	21.99 (20.1, 23.8)	0.908	22.5 (21.0, 24.8)	22.3 (20.4, 24.0)
Diabetes			0.471		
Absence	192 (68.1%)	92 (75.4%)		65 (81.3%)	39 (69.6%)
Presence	90 (31.9%)	30 (24.6%)		15 (18.8%)	17 (30.4%)
Hypertension			0.246		
Absence	170 (60.3)	65 (53.3)		70 (87.5%)	26 (46.4%)
Presence	112 (39.7)	57 (46.7)		10 (12.5%)	30 (53.6%)
TBil (μmol/L)			0.432		
> 20.4	187 (66.3)	78 (63.9)		48 (60.0%)	39 (69.6%)
≤ 20.4	95 (33.7)	44 (36.1)		32 (40.0%)	17 (30.4%)
Albumin (g/L)			0.150		
≥ 35	253 (89.7)	108 (88.5)		70 (87.5%)	47 (83.9%)
< 35	29 (10.3)	14 (11.5)		10 (12.5%)	9 (16.1%)
CA19-9 (U/mL)			0.549		
≤ 100	71 (25.2)	27 (22.1)		24 (30.0%)	33 (58.9%)
> 100	211 (74.8)	95 (77.9)		56 (70.0%)	23 (41.1%)
Adjuvant therapy					
Absence	103 (36.5)	45 (36.9)	0.895	13 (16.3%)	16 (28.6%)
Presence	179 (63.5)	77 (63.1)		67 (83.8%)	40 (71.4%)
AJCC 8th pT stage			0.730		
T1	71 (25.2)	29 (23.8)		12 (15.0%)	13 (23.2%)
T2	184 (65.2)	82 (67.2)		52 (65.0%)	36 (64.3%)
T3	27 (9.6)	11 (9.0)		16 (20.0%)	7 (12.5%)
AJCC 8th pN stage			0.489		
N0	139 (49.3)	60 (49.2)		41 (51.3%)	36 (64.3%)
N1	110 (39.0)	57 (46.7)		31 (38.8%)	18 (32.1%)
N2	33 (11.7)	5 (4.1)		8 (10.0%)	2 (3.6%)
AJCC 8th staging			0.549		
I	128 (45.4)	54 (44.3)		32 (40.0%)	31 (55.4%)
II	121 (42.9)	63 (51.6)		40 (50.0%)	23 (41.1%)
III	33 (11.7)	5 (4.1)		8 (10.0%)	2 (3.6%)
Histologic grade			0.372		
Well/moderately-differentiated	145 (51.4)	59 (48.4)		51 (63.8%)	29 (51.8%)
Ill-differentiated	137 (48.6)	63 (51.6)		29 (36.3%)	27 (48.2%)
Pathological peripheral nerve infiltration			0.521		
Absence	57 (20.2)	22 (18.0)		15 (18.8%)	5 (8.9%)
Presence	225 (79.8)	100 (82.0)		65 (81.3%)	51 (91.1%)
Pathological lymphovascular invasion			0.495		
Absence	191 (67.7)	80 (65.6)		60 (75.0%)	39 (69.6%)
Presence	91 (32.3)	42 (34.4)		20 (25.0%)	17 (30.4%)
Pathological fatty infiltration			0.774		
Absence	65 (23.0)	34 (27.9)		19 (23.8%)	12 (21.4%)
Presence	217 (77.0)	88 (72.1)		61 (76.3%)	44 (78.6%)
OS time (day)^a^	505 (255, 933)	543 (316, 1065)	0.491	479.0 (223.0, 711.5)	684.5 (431.0, 838.5)
RFS time (day)^a^	359 (168, 720)	349 (181, 832)	0.165	384.5 (178.5, 611.0)	492.5 (213.0, 655.0)

*PDAC* pancreatic ductal adenocarcinoma, *BMI* body mass index, *TBil* total bilirubin, *CA19-9* cancer antigen 19-9, *AJCC* American Joint Committee on Cancer

^a^ Data are median (interquartile ranges (IQR))

### Development of the MRI-based Pancreatic Risk Stratification Model (M-PRiSM)

After uni- and multivariable COX regression analysis, CA19-9 (hazard ratio (HR), 1.604; 95% CI: 1.006, 2.559; *p* = 0.047), margin (HR, 1.918; 95% CI: 1.302, 2.825; *p* = 0.001), tumor size (HR, 1.308; 95% CI: 1.099, 1.558; *p* = 0.003), and VER (HR, 0.605; 95% CI: 0.466, 0.786; *p* = 0.047) were associated with OS in PDAC with resection (Table 2).

**Table 2:** Uni- and multivariate analyses for OS based on the development cohort.

Parameters	Univariate analysis					Multivariate analysis				
	Coefficient	HR	95CI_L	95CI_U	*p*-value	Coefficient	HR	95CI_L	95CI_U	*p*-value
Age (years)	0.015	1.015	0.995	1.035	0.139					
Sex										
Female		Reference								
Male	0.198	1.220	0.826	1.800	0.318					
BMI (kg/m²)	0.011	1.011	0.948	1.079	0.730					
Diabetes										
Absence		Reference								
Presence	0.218	1.243	0.834	1.855	0.286					
Hypertension										
Absence		Reference								
Presence	−0.144	0.866	0.584	1.285	0.474					
TBil (μmol/L)										
≤ 20.4		Reference								
> 20.4	−0.084	0.920	0.609	1.388	0.690					
Albumin (g/L)										
≥ 35		Reference								
< 35	0.136	1.146	0.614	2.141	0.669					
CA19-9 (U/mL)										
≤ 100		Reference					Reference			
> 100	0.489	1.631	1.032	2.577	**0.036**	0.473	1.604	1.006	2.559	**0.047**
Location					0.116					
Head		Reference								
Body/tail	−0.139	0.870	0.584	1.297	0.494					
Tumor size (cm)	0.329	1.390	1.191	1.622	**<** **0.001**	0.269	1.308	1.099	1.558	**0.003**
Margin										
Well-defined		Reference					Reference			
Ill-defined	0.721	2.057	1.403	3.015	**<** **0.001**	0.651	1.918	1.302	2.825	**0.001**
SI in T2-weighted images										
Hypointense		Reference								
Iso-/hyperintense	0.390	1.477	0.971	2.246	0.069					
SI in DWI										
Iso-/hypointense		Reference								
Hyperintense	0.350	1.419	0.863	2.334	0.168					
SI on unenhanced T1-weighted imaging										
Iso-/hyperintense		Reference								
Hypointense	0.190	1.210	0.610	2.398	0.586					
SI on arterial phase images										
Iso-/hyperintense		Reference					Reference			
Hypointense	0.887	2.429	1.226	4.811	**0.011**	0.489	1.630	0.763	3.482	0.207
SI on venous phase images										
Iso-/hyperintense		Reference					Reference			
Hypointense	0.497	1.644	1.102	2.455	**0.015**	-0.285	0.752	0.411	1.375	0.355
SI on delayed phase images										
Iso-/hyperintense		Reference					Reference			
Hypointense	0.625	1.868	1.273	2.742	**0.001**	0.244	1.277	0.727	2.243	0.396
MPD dilation										
Absence		Reference								
Presence	−0.372	0.690	0.471	1.010	0.056					
CBD dilation										
Absence		Reference								
Presence	0.086	1.090	0.731	1.626	0.673					
IBD dilation										
Absence		Reference								
Presence	0.046	1.047	0.696	1.576	0.824					
Rim enhancement										
Absence		Reference								
Presence	0.331	1.393	0.950	2.041	0.089					
Peripancreatic fat infiltration										
Absence		Reference								
Presence	0.337	1.400	0.909	2.157	0.127					
Pancreatic atrophy										
Absence		Reference								
Presence	−0.144	0.866	0.577	1.299	0.488					
SMV or PV contact										
Absence		Reference								
Presence	−0.168	0.846	0.463	1.543	0.585					
Cystic degeneration										
Absence		Reference								
Presence	−0.081	0.922	0.581	1.464	0.731					
Hemorrhage					0.244					
Absence		Reference								
Presence	0.387	1.473	0.362	5.992	0.589					
Necrotic										
Absence		Reference								
Presence	0.396	1.486	0.945	2.337	0.086					
Enlarged lymph nodes at MRI										
Absence		Reference					Reference			
Presence	0.594	1.810	1.225	2.675	**0.003**	0.228	1.257	0.831	1.902	0.279
ADC (×10^-3^ mm^2^/s)	−0.055	0.946	0.530	1.691	0.852					
AER	−0.698	0.498	0.364	0.680	**<** **0.001**					
VER	−0.653	0.521	0.399	0.679	**<** **0.001**	-0.502	0.605	0.466	0.786	**<** **0.001**
DER	−0.452	0.636	0.498	0.813	**<** **0.001**					

*PDAC* pancreatic ductal adenocarcinoma, *BMI* body mass index, *TBil* total bilirubin, *CA19-9* cancer antigen 19-9, *DWI* diffusion-weighted imaging, *MPD* main pancreatic duct, *CBD* common bile duct, *IBD* intrahepatic bile duct, *ADC* apparent diffusion coefficient, *PV* portal vein, *SMV* superior mesenteric vein, *AER* arterial enhancement ratio, *VER* venous enhancement ratio, *DER* delayed enhancement ratio

Reference denotes the baseline category in the Cox model

Values in bold denote statistical significance.

**Fig. 2:**
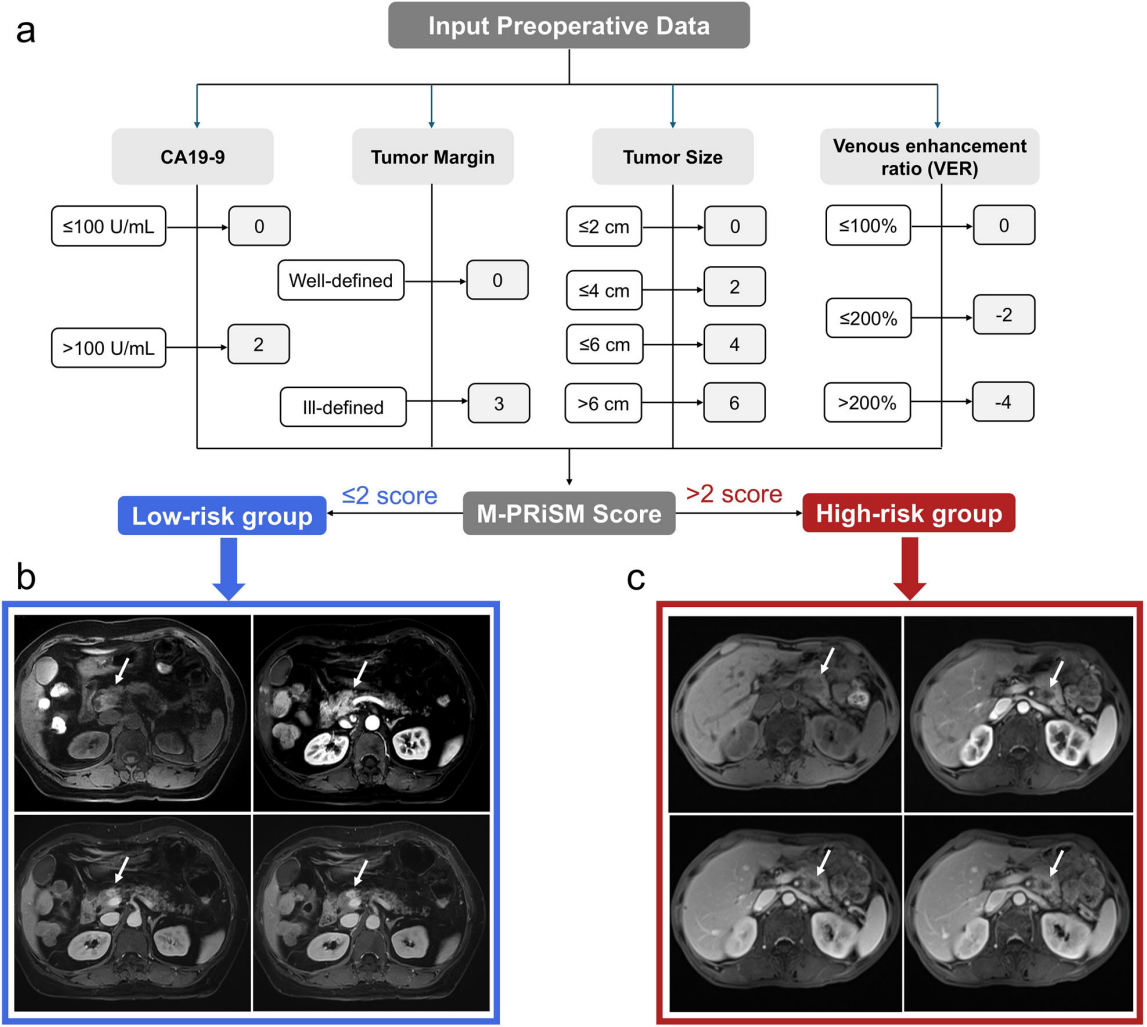
**a** Illustration of MRI-based pancreatic risk stratification model (M-PRiSM) scoring system. **b**, **c** Preoperative axial contrast-enhanced MRI images: unenhanced T1-weighted (upper right), arterial phase (upper left), portal venous phase (lower right), delayed phase (lower left). The arrows represent mass. **b** A 60-year-old female patient with PDAC underwent a radical pancreatectomy. CA19-9 level was 27 U/mL, and the tumor size was approximately 1.3 cm with well-defined margins, and VER was 478%. The patient was classified as low risk (M-PRiSM risk score = −4 points) and survived for 1061 days post-surgery without recurrence. **c** A 63-year-old male patient with PDAC underwent a distal pancreatectomy. CA19-9 level was 148 U/mL, and the tumor size was approximately 2.3 cm with ill-defined margins, and VER was 98%. The patient was classified as high risk (M-PRiSM risk score = 7 points), experienced recurrence 239 days post-surgery, and died after 468 days

The M-PRiSM risk score was developed to predict OS in PDAC patients following surgical resection. This model was constructed using risk factor scores assigned to each parameter (Table 3). The scoring system is derived from variables in the Cox proportional hazards model, including tumor size, margin definition, CA19-9 levels, and VER. The M-PRiSM score thresholds for categorizing patients into low and high-risk groups are set at 2 points according to median score, respectively. For simplicity in calculation and practical use, the M-PRiSM risk score is calculated using the following formula: The M-PRiSM risk score = (tumor size (cm) (≤ 2 = 0, 2–4 = 2, 4–6 = 4, > 6 = 6) + (margin (well-defined = 0; ill-defined = 3))  + (CA19-9 (U/mL) (≤ 37 = 0; > 37 = 2)) +  (VER (%) (> 300 = −4, 200–300 = −2, < 100 = 0) (Table 3). Figure 2 illustrates the preoperative MRI of patients at low- and high-risk levels of OS.

**Table 3:** M-PRiSM score for parameters associated with OS in PDAC

Risk factor	Categories	Score
Tumor size (cm)	≤ 2	0
	> 2 to ≤ 4	2
	> 4 to ≤ 6	4
	> 6	6
Tumor margin	Well-defined	0
	Ill-defined	3
CA19-9 (U/mL)	≤ 100	0
	> 100	2
VER (%)	≤ 100	0
	> 100 to ≤ 200	−2
	> 200	−4

*PDAC* pancreatic ductal adenocarcinoma, *CA19-9* cancer antigen 19-9, *VER* venous enhancement ratio

### Interobserver agreement

Interobserver agreement was good for peripancreatic fat infiltration, enlarged lymph nodes at MRI, hemorrhage, SMV or PV contact, cystic degeneration, SI on arterial phase images, pancreatic atrophy, and ADC, with Kappa/ICC values ranging from 0.653 to 0.808 (95% CI 0.384–0.940). The remaining MRI features showed excellent agreement, with Kappa/ICC values ranging from 0.811 to 0.938 (95% CI: 0.742–0.968) (Table E4).

### Internal and external validation of the M-PRiSM score

**Fig. 3:**
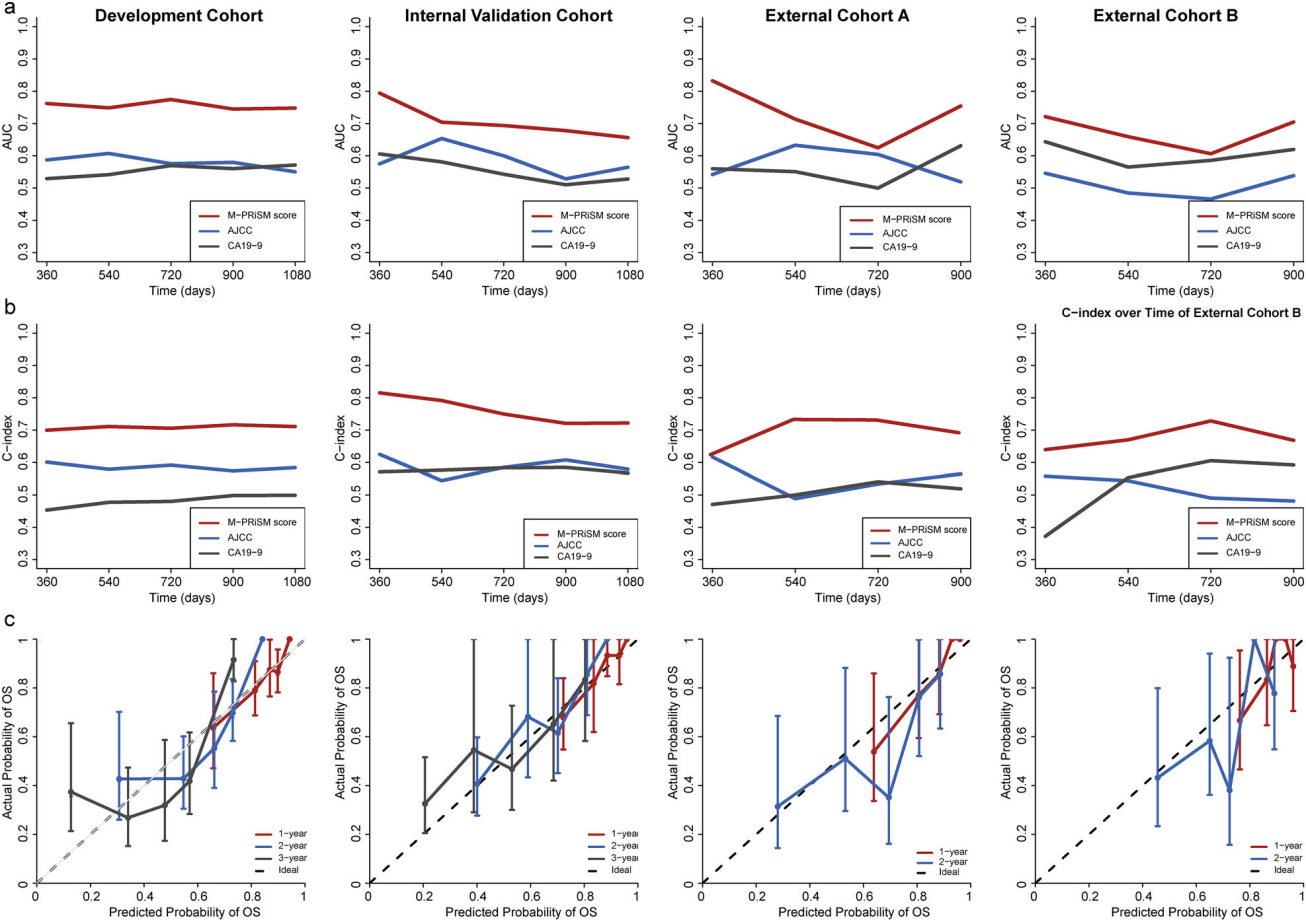
The figures illustrate the discriminative performance of the developed model. **a** The concordance index is shown for the development, internal validation, external validation A, and external validation B cohorts, while the time-dependent areas under the receiver operating characteristic curve (**b**) in the development, internal validation A, and external validation B cohorts assess the model’s discriminatory power at various time points, comparing with major current prognostic factors. **c** The calibration curves assess the model’s calibration performance in the development, internal validation A, and external validation B cohorts at different times. AJCC, American Joint Committee on Cancer

Figure 3a, b displays the C-index and time-dependent AUC for the M-PRiSM risk score across development, internal validation, and two external validation cohorts. The M-PRiSM score showed superior predictive accuracy with C-indexes of 0.726 (95% CI: 0.682–0.770), 0.698 (95% CI: 0.615–0.778), 0.696 (95% CI: 0.610–0.780), and 0.677 (95% CI: 0.561–0.780) in the development, internal validation, external validation A, and B cohorts, respectively, outperforming the 8th AJCC staging (0.498–0.580, all *p* < 0.001) and CA19-9 (0.524–0.606, all *p* < 0.001). The M-PRiSM score also had higher 1- to 5-year AUCs (development: 0.755–0.781; internal validation: 0.659–0.778; external A: 0.712–0.832; external B: 0.658–0.701) compared to AJCC stage (0.435–0.635) and CA19-9 (0.542–0.660) (Table E5). Calibration curves showed strong correlation between predicted and actual OS probabilities, with slopes of 0.74–1.48 (95% CI: −0.68–3.36, *p* = 0.291–0.949) across all cohorts (Fig. 3c and Table E6). In addition, DCA further confirmed the superior clinical utility of the M-PRiSM score compared with AJCC staging and CA19-9, showing greater net benefit across all cohorts (Fig.  E2).

### OS stratification based on M-PRiSM score across various cohorts

**Fig. 4:**
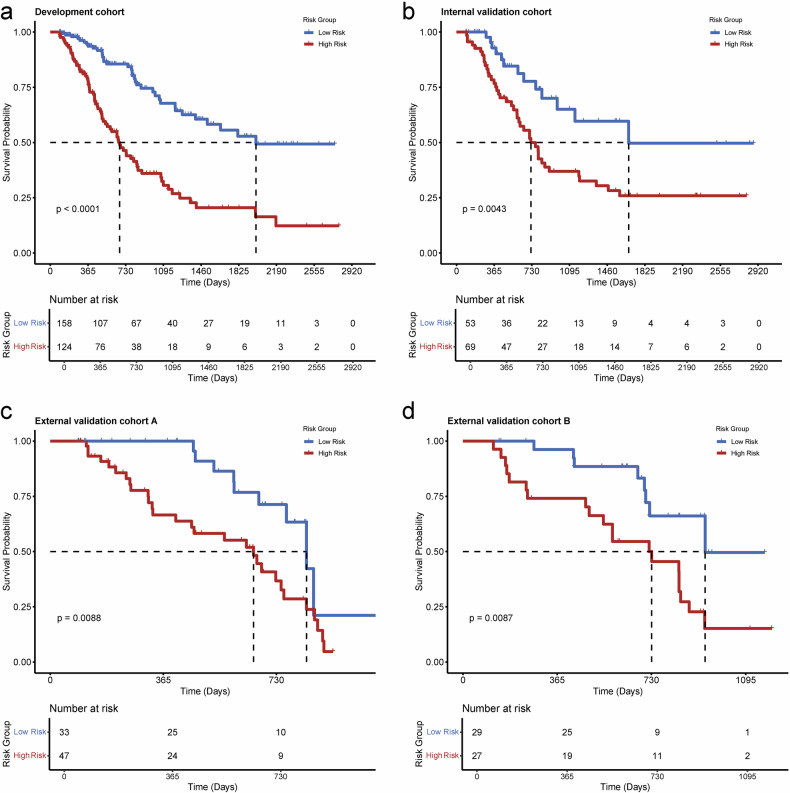
The evaluation of the prognostic stratification ability and potential pathological interpretability of the M-PRiSM risk score in patients with PDAC. Figures show overall survival curves of three prognosis groups based on risk score in (**a**) development, (**b**) internal validation, (**c**) external validation A, and (**d**) external validation B cohorts

Kaplan–Meier curves for the M-PRiSM score stratified OS into low- and high-risk groups across four cohorts (*p* < 0.001, 0.004, 0.009, 0.009, respectively; Fig. 4a–d). 1-year OS probabilities were 93.6%/78.0% (development) and 92.8%/76.8% (internal validation) for low-/high-risk groups. For external cohorts, OS rates over 2–5 years ranged from approximately 50.0% to 20.5% (high-risk) and approximately 85.5% to 49.7% (low-risk). RFS showed similar stratification (Table E7).

### Correlation between M-PRiSM Score and pathologic markers

Our prognostic stratification is closely associated with pathological adverse factors in the development cohort. In the high-risk group, the proportion of poorly differentiated tumors was significantly higher, whereas in the low-risk group, the proportion of poorly differentiated tumors was relatively lower (*p* = 0.026). The incidence of pathological lymphovascular invasion (PLI) and peripheral nerve infiltration was significantly lower in the low-risk group compared to the high-risk group (*p* = 0.003 and *p* = 0.032, respectively), whereas no significant difference was observed for fatty infiltration (*p* = 0.123) (Table E8).

### Survival benefit of adjuvant therapies based on M-PRiSM score

**Fig. 5:**
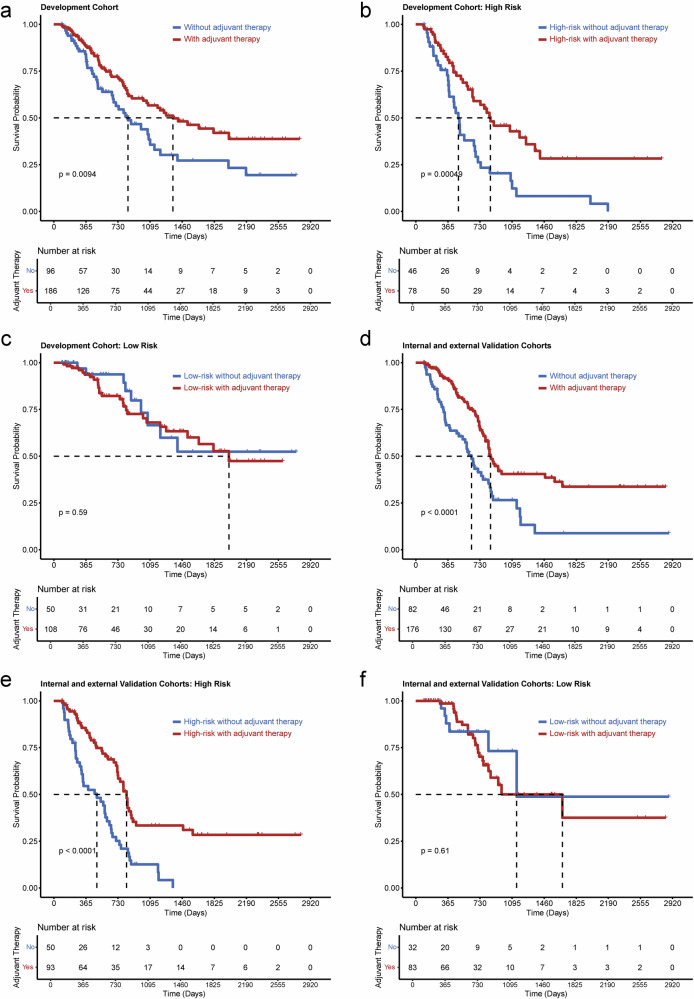
The figures show overall survival outcomes in patients at (**a**) development cohort, (**b**) high-, and (**c**) high-risk subgroups for patients with or without adjuvant therapies. Pictures show overall survival outcomes in patients at (**d**) internal and external cohorts, (**e**) high-, and (**f**) low-risk subgroups for patients with or without adjuvant therapies

In both the development and overall test cohorts, no statistically significant differences were observed in potential clinical and pathological risk characteristics between patients receiving adjuvant therapy and those who did not (all *p* > 0.05, Table E9). In addition, OS was comparable between patients who received adjuvant therapies and those who did not in both the development and overall test cohorts (median OS, 1355 vs. 843 days and 855 vs. 633 days; *p* = 0.009 and < 0.0001). More importantly, when patients were stratified based on the M-PRiSM Score, adjuvant therapies were linked to improved survival outcomes in the high-risk group for OS for both cohorts (median OS, 849 vs. 484 days and 827 vs. 489 days; *p* = 0.0005 and < 0.0001), respectively. Conversely, no significant survival benefit was observed for the low-risk group for both cohorts (*p* = 0.59 and 0.61) (Fig. 5).

### Discussion

Currently, despite the development of preoperatively clinical-radiological prognostic models for predicting postoperative recurrence in PDAC, most are limited by their reliance on CT imaging, lack long-term survival outcomes, single-center designs, and an ambiguous target population, which compromise their clinical reliability and generalizability [18, 19]. In contrast, MRI can capture a broader spectrum of tumor characteristics associated with PDAC prognosis, potentially offering superior prognostic predictive performance [20, 21]. However, there is a scarcity of preoperative MRI-based clinical models for predicting postoperative OS in PDAC. To our knowledge, we are the first to develop and validate an MRI-based pancreatic risk stratification model (M-PRiSM), a robust imaging-based tool built on large-scale, retrospective, multicenter cohorts. This model integrates four easily accessible risk parameters—CA19-9, tumor size, margin, and VER—which are commonly available in clinical practice and MRI examinations.

CA19-9, a key tumor marker for pancreatic cancer, is pivotal in predicting poor prognosis in PDAC, with a sensitivity of 72% and specificity of 86% [22]. Its adverse prognostic impact is linked to its role in forming an immunosuppressive tumor microenvironment, angiogenesis, macrophage polarization, and extracellular matrix remodeling [23]. Additionally, CA19-9 upregulation by tumor cells and antigen-presenting cancer-associated fibroblasts (apCAFs) is associated with increased cellular growth, invasion, and metastasis [24]. Given that 10–40% of patients may not express CA19-9, combining this biomarker with others is crucial to enhance diagnostic accuracy and achieve more reliable outcomes. In addition, our findings confirm tumor size on MRI as an independent prognostic factor for postoperative OS, consistent with prior studies [25, 26]. Moreover, our findings indicated that an ill-defined tumor margin on MRI was associated with poorer OS in PDAC patients, potentially reflecting more aggressive biological characteristics [27, 28]. Notably, we identified a negative correlation between VER and OS; lower VER values were associated with worse prognosis. The imaging density/signal and enhancement variations of the tumor may be associated with its biological characteristics. For example, low density in the venous phase on CT is closely related to postoperative recurrence in PDAC, which may be related to lower tumor cell density, more intratumoral necrosis, and stromal fibrosis [29]. A recent study with a small-sample cohort demonstrated that SER_lt_ea_ (representing the signal enhancement ratio of the tumor on multi-phase contrast-enhanced-MRI) correlates with tumor stroma/epithelium, indirectly reflecting PDAC prognosis [30]. Compared to the qualitative assessment of venous phase signal intensity, VER more accurately reflects the contrast uptake of tumor tissue relative to normal tissue, offering better biological insight. Additionally, this simple quantitative metric has shown generalizability and operability in our multicenter validation cohorts, potentially mitigating the impact of different imaging equipment, scanning parameters, and patient variations. Moreover, compared with radiomics and deep learning-based models, which often rely on high-dimensional features and complex algorithms, this score is easier to interpret, simpler to implement, and more adaptable to routine clinical workflows, enhancing its practical applicability.

The M-PRiSM score we developed effectively and reliably predicts postoperative OS, with C-indexes of 0.73, 0.70, 0.70, and 0.68 across development, internal validation, and two external validation cohorts, respectively. In the internal validation cohort, the time-dependent AUC values ranged from 0.66 to 0.80 for 1- to 4-year periods, demonstrating robust predictive performance. Similarly, in two external validation cohorts, the model’s predictive performance was confirmed, with time-dependent AUCs ranging from 0.69 to 0.83 across 1- and 2-year follow-up intervals. These findings collectively support the model’s generalizability and robustness across diverse cohorts. Notably, the M-PRiSM score demonstrated stable performance among validation sets, outperforming currently recognized postoperative risk indicators for PDAC (the 8th AJCC staging and CA19-9). According to the M-PRiSM score, patients are stratified into low- and high-risk categories. Interestingly, the M-PRiSM score not only facilitates effective risk stratification for OS at 1 to 5 years follow-ups but also exhibits similar effectiveness in stratifying postoperative RFS. Moreover, risk stratification exhibited strong correlations with critical pathological features, including tumor differentiation, PLI, and perineural invasion (PNI), which are major determinants of postoperative prognosis in PDAC. This association provides a histopathological basis for the interpretability of our M-PRiSM model, addressing the common limitation of insufficient clinical credibility in such predictive models.

Adjuvant therapies are assuming a crucial role in the management of pancreatic cancer [31]. Although a substantial proportion of patients with PDAC achieved improved survival through postoperative adjuvant therapy, they also frequently experience chemotherapy toxicity or treatment insensitivity. This finding underscores the need for clinicians to identify patients likely to derive greater survival benefits from adjuvant therapy, thereby minimizing adverse effects, optimizing treatment application, and refining follow-up strategies [32, 33]. Following risk stratification based on the M-PRiSM score, patients in the high-risk group showed improved survival benefits from adjuvant therapy, whereas those in the intermediate and low-risk groups did not. These findings suggest that M-PRiSM-based risk stratification could serve as a valuable tool for guiding adjuvant therapy decisions and precision care in PDAC. For example, as shown in Fig. 2, a high-risk patient with 7 points (M-PRiSM score) developed early recurrence and died within 15 months, while a low-risk patient with −4 points remained recurrence-free for over 3 years. This contrast illustrates the value of M-PRiSM in guiding individualized postoperative management.

Our study has several limitations. First, the retrospective design inherently carries some degree of bias. Additionally, the model was developed at a high-volume surgical center with potentially more specialized surgical expertise and medical conditions, which may lead to differences in patient outcomes. Moreover, the external validation cohort had a relatively small sample size and limited follow-up duration, which, to some extent, restricts the model’s generalizability. Furthermore, the variability in MRI acquisition protocols across centers represents a limitation that may affect the consistency and generalizability of imaging-derived biomarkers (such as ADC). In addition, the current model is more suitable for guiding personalized postoperative adjuvant treatment strategies, while its role in the neoadjuvant setting requires validation in larger studies. Future research should focus on validating the model’s performance in larger, long-term, and multicenter external datasets with standardized imaging protocols and applying harmonization techniques to reduce variability.

In summary, the M-PRiSM model, incorporating CA19-9, tumor margin, tumor size, and VER, offers superior predictive performance for postoperative OS in PDAC compared to existing models and offers robust pathological interpretability. Stratifying patients into prognostic risk groups identifies those at higher risk who may benefit most from adjuvant therapies. This novel MRI-based preoperative tool improves prognostic accuracy, supports personalized treatment decisions, and complements current staging systems and prognostic indicators.

## Supplementary information

ELECTRONIC SUPPLEMENTARY MATERIAL

## Data Availability

The datasets used and/or analyzed during the current study are available from the corresponding author upon reasonable request.

## Abbreviations

ADC: Apparent diffusion coefficient
AER: Arterial enhancement ratio
AJCC: American Joint Committee on Cancer
AUC: Area under the curve
BMI: Body mass index
CA19-9: Cancer antigen 19-9
DCA: Decision curve analysis
DER: Delayed enhancement ratio
IBD: Intrahepatic bile duct
MPD: Main pancreatic duct
OS: Overall survival
PDAC: Pancreatic ductal adenocarcinoma
PLI: Pathological lymphovascular invasion
PV: Portal vein
RFS: Recurrence-free survival
ROC: Receiver operating characteristic
SI: Signal intensity
SMV: Superior mesenteric vein
TBil: Total bilirubin
VER: Venous enhancement ratio
