# Supplementary material for: MRI-based risk stratification for predicting overall survival in pancreatic ductal adenocarcinoma
Source: Insights Imaging. 2025 Sep 24;16:205. doi: 10.1186/s13244-025-02088-1 (PMC12460861; doi:10.1186/s13244-025-02088-1)
Supplement: Supplementary file 1 — ELECTRONIC SUPPLEMENTARY MATERIAL [file 13244_2025_2088_MOESM1_ESM.pdf]

MRI-Based Risk Stratification for Predicting Overall Survival in Pancreatic Ductal Adenocarcinoma

ELECTRONIC SUPPLEMENTARY MATERIAL

Appendix S1

Detailed Imaging Acquisition

All contrast-enhanced MRIs were conducted using one of the following MRI systems: [MAGNETOM Aera), [3.0T Discovery MR750), [MEDICAL SYSTEMS SIGNA Architect), [Ingenia CX 3.0T). Contrast agents used included gadolinium diethylenetriamine pentaacetic acid (Gd-DTPA; Magnevist), gadobenate dimeglumine, or gadobenate dimeglumine, which reached the ascending aorta following intravenous injection. For acquisition of Gd-DTPA, 0.1 mmol/kg of gadolinium diethylenetriamine pentaacetic acid, gadobenate dimeglumine, or gadobenate dimeglumine was injected at a rate of 1.0–2.0 mL/s.

Patients were instructed to fast for 6–8 hours prior to their MRI scans. The MRI protocols comprised: (a) T2-weighted imaging; (b) diffusion-weighted imaging with b values of 0 and 500 s/mm² [(MAGNETOM Aera); (Amira, Siemens), (Ingenia)], 0, 50, and 800 s/mm² [MEDICAL SYSTEMS SIGNA Architect), or 0, and 800 s/mm² [Philips Ingenia CX 3.0T), [3.0T Discovery MR750) with apparent diffusion coefficient maps generated using the monoexponential model; (c) in-phase and out-of-phase T1-weighted imaging; and (d) dynamic T1-weighted imaging during the precontrast phase, arterial phase (20-30 seconds post-contrast injection), portal venous phase (60-70 seconds post-contrast injection), and delayed phase (160–180 seconds post-contrast injection).

Appendix S2

Detailed Explanations for Imaging Characteristics

A well-defined margin is characterized by over 75% of the lesion having a clear and discrete boundary, while an ill-defined margin is identified when the lesion fails to meet this criterion(1). Tumor size was measured as the lesion’s greatest diameter in the axial plane; Rim enhancement was defined irregular ringlike enhancement with a relatively hypovascular central area on dynamic-enhanced images(2). Enlarged lymph nodes at imaging referred to lymph nodes that are larger than normal, often greater than 1 cm in short-axis diameter. Main pancreatic duct (MPD) dilation was identified when the maximum diameter of the main pancreatic duct exceeded 2 mm(3). Common bile duct (CBD) dilation was considered present if the short-axis diameter of the common bile duct was greater than 7 mm in individuals under 60 years of age or 9 mm in those 60 years or older(3). The intrahepatic bile duct (IBD) diameters were measured to identify dilation, with thresholds of 3 mm and greater indicating dilation (4). Pancreatic atrophy was indicated by a ratio of the main pancreatic duct width to total pancreatic parenchymal width of less than 0.50(4). Peripancreatic fat infiltration was characterized as the presence of haziness or strand structures derived from the tumor (5). Cystic degeneration was characterized as the formation of cyst-like areas within the tumor that are not attached to the pancreatic duct, which appear as high signal intensity on T2-weighted images, with no contrast enhancement on dynamic-enhanced images. Necrosis on MRI was identified when the lesion exhibited an irregular peripheral region with high signal intensity on T2-weighted images; otherwise, it was classified as absent(6). SMV or PV contact was defined as the tumor has contact with SMV or PV, but the sector angle of the contact surface does not exceed 180°.

Appendix S3 Detailed Statistical Analysis

Appendix S3.1

To assess inter-reader agreement, Cohen's kappa was used for categorical variables, while intraclass correlation was applied for continuous variables. Agreement was considered poor (κ or ICC: 0.00–0.20), fair (κ or ICC: 0.21–0.4), moderate (κ or ICC: 0.41–0.6), good (κ or ICC: 0.61–0.8), or excellent (κ or ICC > 0.8).

Appendix S3.2

We applied a backward stepwise selection process to refine the model, sequentially removing non-significant variables until all remaining predictors were significant. To address potential collinearity issues, we computed the correlation matrix of the factors and generated a heatmap to identify highly correlated predictors. This step helped to avoid collinearity problems in the model. During each step of the variable selection process, the Akaike Information Criterion (AIC) was used to assess model performance, with a lower AIC indicating a better fit while accounting for model complexity.

Prior to conducting multivariate analysis, pairwise correlation analysis was performed for collinearity diagnosis. Correlations greater than 0.7 or less than -0.7 were considered significant, indicating potential collinearity among factors. These relationships were visualized using a correlation heatmap.

Appendix S3.3

Internal validation was performed using bootstrapping with 1000 iterations to estimate model performance. To address potential overfitting, optimism-adjusted C-index and AUC were calculated to provide a more accurate performance estimate. The calibration accuracy of the risk score was evaluated using a calibration plot and calibration slope, involving a comparative analysis of predicted values against observed outcomes. Additionally, a chi-square test was employed to assess the correlation between risk groups and pathological factors.

Table E1: Detailed parameters of MRI sequences in Zhongshan Hospital, Fudan University

Center	MRI	Parameters	TR	TE	FOV	Matrix	Section thickness(mm)	Gap
Zhongshan Hospital Fudan University	1.5T MR (MAGNETOM Aera, Siemens)	T2-weighted imaging	4918	106	285×380	384×273	5.5	1.2
		DWI [a]	5100	55	285×380	192×154	5.5	1.2
		T1-weighted in-phase and opposed-phase imaging	6.88	2.39/4.77	356×380	320×240	3.5	0
		Contrast-enhanced T1-weighted imaging	3.47	1.36	308×380	320×240	3	0
	3.0T MR (Prisma, Siemens)	T2-weighted imaging	587	103	380×344	320×320	6	1.2
		DWI [a]	5000	65	380×310	130×130	5	1.2
		T1-weighted in-phase and opposed-phase imaging	3.97	1.29/2.52	380×285	320×288	3.0	0
		Contrast-enhanced T1-weighted imaging	3.10	1.23	380×309	320×288	3	0

Note. — TR = Repetition time; TE = Echo time; FOV = Field of view;
DWI = Diffusion-weighted Imaging.

[a] performed with b values of 0 and 500 sec/mm^2

Table E2: Detailed parameters of MRI sequences in multi-centers

Center	MRI	Parameters	TR	TE	FOV	Matrix	Section thickness(mm)	Gap
First Affiliated Hospital of Bengbu Medical University (center 2)	Philips Ingenia CX 3.0T PHILIPS	T2-weighted imaging	1250	70	36.2×36.2	212×216	5	0.5
		DWI[b]	3000	59.3	37.5×37.5	132×161	6.5	3.25
		T1-weighted in-phase and opposed-phase imaging	165	2.3/1.15	34.8×34.8	152×194	7	0
		Contrast-enhanced T1-weighted imaging	3.03	1.42	34.5×34.5	232×264	4.6	0
Affiliated Hospital of Jiangnan University (center 3)	GE MEDICAL SYSTEMS SIGNA Architect 3.0T	T2-weighted imaging	10000	91.9	38×38	288×288	6.0	2.0
		DWI[c]	8000	68.4	38×38	128×130	6.0	2.0
		T1-weighted in-phase and opposed-phase imaging	4.8	2.2/1.1	38×30.4	288×224	2.5	0

		Contrast-enhanced T1-weighted imaging	4.8	1.7	38×30.4	288×224	2.6	0
	Philips Ingenia CX 3.0T	T2-weighted imaging	22552	89.1	40×40	288×58	6.0	1.0
		DWI[c]	14452	59.3	36.5×42.9	136×128	5.0	1.5
		T1-weighted in-phase and opposed-phase imaging	3.63	2.4/1.2	40×31.4	236×194	2.0	0
		Contrast-enhanced T1-weighted imaging	3.23	1.7	40×35.2	268×251	2.0	0
The First Hospital of Jiaxing (center 4)	GE3.0T Discovery MR750	T2-weighted imaging	12000	80.5	40×40	352×352	6.5	1.0
		DWI [b]	6666.7	47.4	40×40	128×128	6.5	1.0
		T1-weighted in-phase and opposed-phase imaging	4	2.29/1.12	40×40	320×320	5	2.5
		Contrast-enhanced T1-weighted imaging	3.76	1.67	40×40	256×320	3	0
	Siemens Area1.5T	T2-weighted imaging	8941.1	85.0	35×35	256×256	6	1.2

		DWI [b]	5200	67	29.7×38	240×384	7	1.4
		T1-weighted in-phase and opposed-phase imaging	200	4.9/2.4	31.0×38.1	182×320	6.5	1.3
		Contrast-enhanced T1-weighted imaging	5.7	2.2	38×38	224×320	3	0
Xiamen Branch of Zhongshan Hospital (center 5)	1.5T MR (Amira, Siemens)	T2-weighted imaging	2000	85	390×390	325×325	5.5	1.1
		DWI [a]	4200	55	400×275	267×183	5.5	1.1
		T1-weighted in-phase and opposed-phase imaging	140	2.38/4.77	380×285	633×475	5.5	1.1
		Contrast-enhanced T1-weighted imaging	3.92	1.46	390×305	325×254	3	0.6
	3.0T MR (Ingenia, Philips)	T2-weighted imaging	8365	90	390×390	300×300	5	1
		DWI [a]	3368	67.5	360×310	120×90	5	1
		T1-weighted in-phase and opposed-phase imaging	180	1.15/2.3	400×310	224×171	5	1

		Contrast-enhanced T1-weighted imaging	3.1	1.11	390×340	228×200	5	-2.5

Note. — TR = Repetition time; TE = Echo time; FOV = Field of view;
DWI = Diffusion-weighted Imaging;

[a] performed with b values of 0 and 500 sec/mm^2

[b] performed with b values of 0, 800 sec/mm^2

[c] performed with b values of 0, 50, 800 sec/mm^2

Table E3. Baseline radiological characteristics

**Clinicopathological Characteristics**	Development (n=282)	Internal validation cohort (n=122)	*P* value	External validation A (n=80)	External validation B (n=56)
Location			0.513		
Head	178 (63.1)	72 (58.2)		58 (72.5%)	32 (57.1%)
Body/tail	104 (36.9)	50 (41.8)		22 (27.5%)	24 (42.9%)
Tumor size (cm) *	2.7 (2.1,3.4)	2.7(2.2,3.4)	0.160	2.9 (2.2, 3.7)	2.8 (2.3, 3.4)
Margin			0.09		
Well-defined	176 (62.4)	61 (50.0)		37 (46.3%)	31 (55.4%)
Ill-defined	106(37.6)	61 (50.0)		43 (53.8%)	25 (44.6%)
Signal in T2-weighted images			0.894		
Hypointense	98 (34.8)	42 (34.4)		19 (23.8%)	20 (35.7%)
Iso-/hyper-intense	184 (65.2)	80 (65.6)		61 (76.3%)	36 (64.3%)
Signal in DWI			0.291		
Iso-/hypointense	76 (27.0)	25 (20.5)		8 (10.0%)	11 (19.6%)
Hyperintense	206 (73.0)	97 (79.5)		72 (90.0%)	45 (80.4%)
Signal on T1-weighted			0.256		

precontrast images					
Iso-/hyperintense	31 (11.0)	8 (6.6)		2 (2.5%)	4 (7.1%)
Hypointense	251 (89.0)	114 (93.4)		78 (97.5%)	52 (92.9%)
Signal on arterial phase images			0.150		
Iso-/hyperintense	49 (17.4)	14 (11.5)		5 (6.3%)	1 (1.8%)
Hypointense	233 (82.6)	108 (88.5)		75 (93.8%)	55 (98.2%)
Signal on venous phase images			0.600		
Iso-/hyperintense	119 42.2)	44 (36.1)		15 (18.8%)	13 (23.2%)
Hypointense	163 (57.8)	78 (63.9)		65 (81.3%)	43 (76.8%)
Signal on delayed phase images			0.700		
Iso-/hyperintense	165 (58.5)	67 (54.9)		26 (32.5%)	29 (51.8%)
Hypointense	117 (41.5)	55 (45.1)		54 (67.5%)	27 (48.2%)
MPD dilation			0.798		
Absence	113 (40.1)	55 (45.1)		31 (38.8%)	19 (33.9%)
Presence	169 (59.9)	67 (54.9)		49 (61.3%)	37 (66.1%)
CBD dilation			0.09		
Absence	184 (65.2)	69 (56.6)		55 (68.8%)	37 (66.1%)

Presence	98 (34.8)	53 (43.4)		25 (31.3%)	19 (33.9%)
IBD dilation			0.288		
Absence	187 (66.3)	74 (60.7)		55 (68.8%)	38 (67.9%)
Presence	95 (33.7)	48 (39.3)		25 (31.3%)	18 (32.1%)
Rim enhancement					
Absence	146 (51.8)	49 (40.2)		48 (60.0%)	27 (48.2%)
Presence	136 (48.2)	73 (59.8)		32 (40.0%)	29 (51.8%)
Peripancreatic fat infiltration			0.471		
Absence	76 (27.0)	35 (28.7)		35 (43.8%)	19 (33.9%)
Presence	206 (73.0)	87 (71.3)		45 (56.3%)	37 (66.1%)
Pancreatic atrophy			0.362		
Absence	156 (55.3)	70 (57.4)		39 (48.8%)	27 (48.2%)
Presence	126 (44.7)	52 (42.6)		41 (51.3%)	29 (51.8%)
SMV or PV contact			0.731		
Absence	236 (83.7)	101 (82.7)		56 (70.0%)	48 (85.7%)
Presence	46 (16.3)	21 (17.2)		24 (30.0%)	8 (14.3%)
Enlarged lymph nodes at MRI			0.671		
Absence	204 (72.3)	86 (70.5)		72 (90.0%)	47 (83.9%)

Presence	78 (27.7)	36 (29.5)		8 (10.0%)	9 (16.1%)
Cystic degeneration			0.528		
Absence	220 (78.0)	99 (81.1)		54 (67.5%)	45 (80.4%)
Presence	62 (22.0)	23 (18.9)		26 (32.5%)	11 (19.6%)
Hemorrhage			0.316		
Absence	277 (98.2)	121 (99.2)		79 (98.8%)	56 (100.0%)
Presence	5 (1.8)	1 (0.8)		1 (1.3%)	0 (0.0%)
Necrosis			0.885		
Absence	215 (76.2)	89 (73.0)		63 (78.8%)	48 (85.7%)
Presence	67 (23.8)	33 (27.0)		17 (21.3%)	8 (14.3%)
ADC ($\times 10^{-3}$ mm^2/s) *	1.45 (1.27,1.70)	1.43 (1.21,1.67)		1.03 (0.91,1.25)	1.44 (1.26,1.61)
AER*	0.97(0.52,1.40)	0.76(0.42,1.34)	0.165	0.73 (0.31, 1.24)	0.75 (0.49, 1.02)
VER*	1.61(1.09,2.11)	1.48(1.00,2.01)	0.167	1.51 (0.85, 2.22)	1.40 (1.05, 1.87)
PER*	1.87(1.34,2.38)	1.69(1.26,2.24)	0.178	1.77 (1.18, 2.51)	1.66 (1.40, 2.07)

Note.- Unless specified otherwise, data are presented as the number of patients, with percentages in parentheses.

*Data are shown as medians, with the interquartile range in parentheses.

DWI = diffusion-weighted imaging, MPD = main pancreatic duct, CBD = common bile duct, IBD = Intrahepatic bile duct, DWI = Diffusion-weighted Imaging, AER=arterial enhancement ratio, PV = portal vein, SMV = superior mesenteric vein, ADC = apparent diffusion coefficient, AER=arterial enhancement ratio, VER=venous enhancement ratio, DER= delayed enhancement ratio

Table E4: Interobserver agreement of MRI features in PDAC

MR feature	Kappa value/ICC value	95% CI Lower	95% CI Upper
SI on unenhanced T1-weighted imaging *	0.827	0.742	0.912
Margin *	0.841	0.795	0.887
Rim enhancement *	0.811	0.762	0.860
SI in T2WI	0.846	0.798	0.894
SI in DWI*	0.829	0.773	0.885
SI on arterial phase images *	0.788	0.710	0.865
SI on venous phase images *	0.844	0.796	0.892
SI on delayed phase images *	0.815	0.766	0.864
CBD dilation *	0.832	0.785	0.880
IBD dilation *	0.918	0.883	0.953
MPD dilation*	0.938	0.909	0.968
SMV or PV contact	0.743	0.671	0.816
Pancreatic atrophy *	0.783	0.730	0.836
Peripancreatic fat infiltration *	0.653	0.588	0.717
Necrosis*	0.837	0.783	0.892
Cystic degeneration*	0.750	0.680	0.819
Enlarged lymph nodes at MRI*	0.668	0.597	0.739
Hemorrhage *	0.662	0.384	0.940
ADC ($\times 10^{-3}$ mm^2/s) †	0.794	0.761	0.823
AER(%)†	0.816	0.785	0.842
VER(%)†	0.827	0.799	0.852
DER(%)†	0.808	0.776	0.835
Tumor Size†	0.882	0.862	0.900

Note- PDAC = pancreatic ductal adenocarcinoma; SI = signal intensity; ICC = Intraclass Correlation Coefficient; MPD = main pancreatic duct, CBD = common bile duct, IBD = Intrahepatic bile duct; ADC = apparent diffusion coefficient, PV = portal vein, SMV = superior mesenteric vein, AER=arterial enhancement ratio, VER=venous enhancement ratio, DER= delayed enhancement ratio

* κ coefficient. † Intraclass correlation coefficient

Table E5. Comparison capacity of the MPRSM Score, the 8th AJCC staging and CA19-9 for predicting postoperative OS

		Development						Internal Validation			External Validation A			External Validation B		
		Apparent			Optimism-corrected			Apparent			Apparent			Apparent		
		mean	lower	upper	mean	lower	upper	mean	lower	upper	mean	lower	upper	mean	lower	upper
M-PRiSM	C-index	0.73	0.68	0.77	0.73	0.67	0.78	0.70	0.62	0.78	0.70	0.61	0.78	0.68	0.56	0.78
	1-year AUC	0.75	0.67	0.83	0.76	0.66	0.84	0.80	0.68	0.91	0.83	0.72	0.94	0.74	0.49	0.94
	2-year AUC	0.79	0.72	0.85	0.79	0.72	0.85	0.72	0.62	0.83	0.70	0.56	0.84	0.69	0.53	0.83
	3-year AUC	0.75	0.66	0.84	0.75	0.66	0.84	0.64	0.52	0.77		NA			NA	
	4-year AUC	0.71	0.60	0.82	0.72	0.60	0.83	0.66	0.53	0.80		NA			NA	
	5-year AUC	0.70	0.58	0.83	0.72	0.58	0.85	0.56	0.38	0.74		NA			NA	
the 8th AJCC staging	C-index	0.58	0.51	0.63		NA		0.58	0.51	0.64	0.57	0.49	0.65	0.50	0.38	0.61
	1-year AUC	0.60	0.50	0.70		NA		0.59	0.47	0.70	0.56	0.42	0.69	0.55	0.34	0.76
	2-year AUC	0.58	0.50	0.66		NA		0.59	0.48	0.69	0.64	0.43	0.79	0.52	0.36	0.67
	3-year AUC	0.55	0.46	0.63		NA		0.56	0.44	0.68		NA			NA	

	4-year AUC	0.54	0.45	0.64		NA		0.54	0.41	0.67		NA			NA	
	5-year AUC	0.50	0.40	0.61		NA		0.59	0.41	0.76		NA			NA	
CA19-9	C-index	0.53	0.49	0.58		NA		0.55	0.50	0.60	0.52	0.43	0.61	0.61	0.50	0.71
	1-year AUC	0.51	0.43	0.60		NA		0.61	0.54	0.68	0.56	0.40	0.68	0.64	0.43	0.83
	2-year AUC	0.57	0.51	0.64		NA		0.55	0.46	0.64	0.54	0.40	0.70	0.66	0.51	0.81
	3-year AUC	0.57	0.50	0.65		NA		0.52	0.42	0.61		NA			NA	
	4-year AUC	0.58	0.49	0.67		NA		0.47	0.37	0.57		NA			NA	
	5-year AUC	0.58	0.48	0.69		NA		0.48	0.36	0.61		NA			NA	

Table E6 The calibration slopes in different groups

slope	Development cohort	P value	Internal validation cohort	P value	External validation A cohort	P value	External validation B cohort	P value
**1-year**	1.18 (0.66, 1.69)	0.865	1.33 (0.90, 1.78)	0.746	1.48 (1.10, 1.87)	0.750	1.47 (-0.42, 3.36)	0.893
**2-year**	0.99(-0.17, 2.15)	0.949	1.12 (0.39, 1.86)	0.388	0.81 (-0.27, 1.90)	0.291	1.02 (-1.04, 3.10)	0.439
**3-year**	0.80(-0.68, 2.28)	0.919	0.74 (0.17, 1.32)	0.380	*NA*	*NA*	*NA*	*NA*

Table E7 OS and RFS in stratified cohorts

Cohorts	M-PRiSM-stratified cohorts	1-year	2-year	3-year	4-year	5-year
OS						
Development	High Risk	78.0	46.4	30.6	20.5	20.5
Development	Low Risk	93.6	85.5	67.8	60.6	52.8
Internal Validation	High Risk	76.8	50.0	36.9	30.4	25.9
Internal Validation	Low Risk	92.8	77.7	65.0	59.6	49.7
External Validation A	High Risk	66.6	36.7	NA	NA	NA
External Validation A	Low Risk	100.0	71.3	NA	NA	NA
External Validation B	High Risk	74.1	50.0	NA	NA	NA
External Validation B	Low Risk	96.2	66.1	NA	NA	NA
RFS						
Development	High Risk	48.9	26.1	18.7	14.5	9.7
Development	Low Risk	78.7	55.5	47.8	37.7	33.0
Internal Validation	High Risk	49.4	33.1	30.9	28.5	22.2
Internal Validation	Low Risk	78.1	54.4	46.0	35.8	35.8
External Validation A	High Risk	55.7	18.9	NA	NA	NA
External Validation A	Low Risk	84.7	58.7	NA	NA	NA
External Validation B	High Risk	62.1	20.8	NA	NA	NA
External Validation B	Low Risk	74.3	33.1	NA	NA	NA

Table E8 Correlation between M-PRiSM-derived risk groups and pathologic markers

Variable	Category	Low Risk	High Risk	P value
Histologic grade	Well/moderately-differentiated	91 (57.6%)	54 (43.5%)	0.026
	Ill-differentiated	67 (42.4%)	70 (56.5%)	
Pathological lymphovascular invasion	Absence	118 (74.7%)	71 (57.3%)	0.003
	Presence	40 (25.3%)	53 (42.7%)	
Pathological peripheral nerve infiltration	Absence	31 (19.6%)	12 (9.7%)	0.032
	Presence	127 (80.4%)	112 (90.3%)	
Pathological fatty infiltration	Absence	37 (23.4%)	19 (15.3%)	0.123
	Presence	121 (76.6%)	105 (84.7%)	

Note.—Unless stated otherwise, data are presented as count (percentages)

Table E9 Patient Characteristics for adjuvant therapy in development and overall test cohorts

		Development cohort			Overall test cohort		
		No Adjuvant Therapy (N=96)	Adjuvant Therapy (N=186)	P value	No Adjuvant Therapy (N=82)	Adjuvant Therapy (N=176)	P value
Tumor size (cm)		2.90 [2.20, 3.23]	2.60 [2.10, 3.50]	0.88	2.77 [2.20, 3.50]	2.80 [2.20, 3.50]	0.759
CA19-9 (U/mL)	≤ 37	25 (26.0)	46 (24.7)	0.924	23 (28.0)	61 (34.7)	0.362
	> 37	71 (74.0)	140 (75.3)		59 (72.0)	115 (65.3)	
Histologic grade	Well/moderately-differentiated	51 (53.1)	94 (50.5)	0.775	37 (45.1)	101 (57.4)	0.088
	Ill-differentiated	45 (46.9)	92 (49.5)		45 (54.9)	75 (42.6)	
Pathological lymphovascular invasion	Absence	63 (65.6)	126 (67.7)	0.822	55 (67.1)	132 (75.0)	0.239
	Presence	33 (34.4)	60 (32.3)		27 (32.9)	44 (25.0)	
Pathological peripheral nerve infiltration	Absence	12 (12.5)	31 (16.7)	0.455	11 (13.4)	29 (16.5)	0.654
	Presence	84 (87.5)	155 (83.3)		71 (86.6)	147 (83.5)	
Pathological fatty infiltration	Absence	14 (14.6)	42 (22.6)	0.151	18 (22.0)	45 (25.6)	0.635
	Presence	82 (85.4)	144 (77.4)		64 (78.0)	131 (74.4)	

AJCC 8th staging	I	45 (46.9)	83 (44.6)	0.87	33 (40.2)	84 (47.7)	0.527
	II	41 (42.7)	80 (43.0)		44 (53.7)	82 (46.6)	
	III	10 (10.4)	23 (12.4)	0.88	5 (6.1)	10 (5.7)	

Note.—Unless stated otherwise, data are presented as median (interquartile range) or count (percentages)
Overall test cohort included internal validation, external validation A, and external validation B cohort.

Figure E1

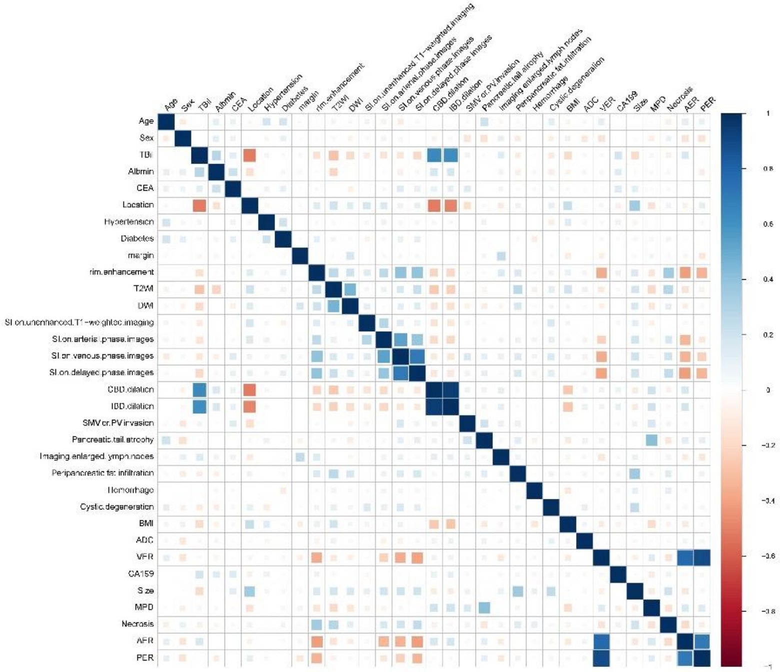

Fig E1. This heatmap illustrates the collinearity among various parameters.

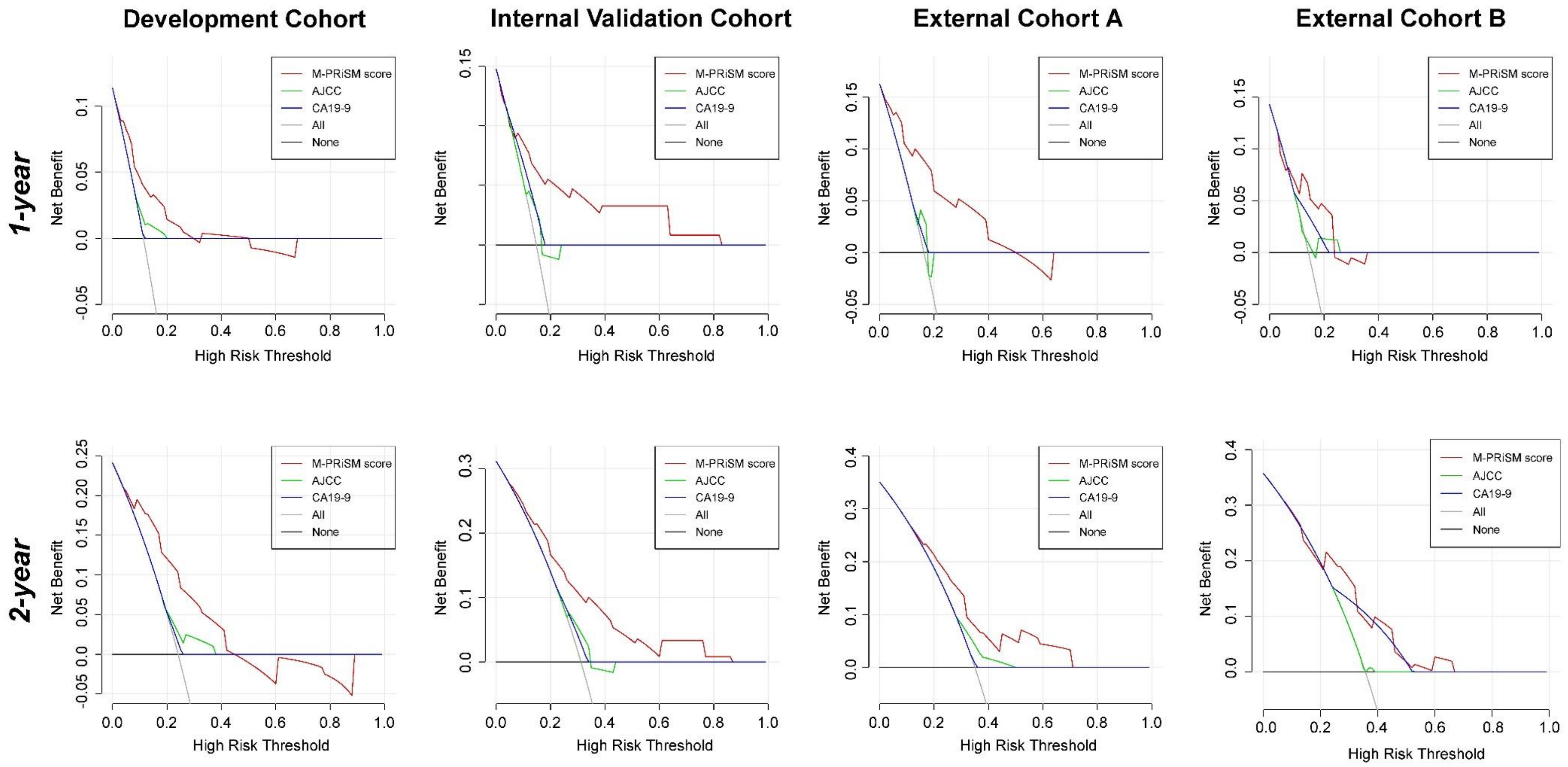

Fig E2. Decision curve analysis (DCA) comparing the net clinical benefit of the M-PRiSM score, AJCC staging, and CA19-9 for predicting 1-year and 2-year postoperative overall survival in four cohorts: Development, internal validation, external cohort A, and external cohort B.

References:

1. Shin N, Kang TW, Min JH, et al. Utility of Diffusion-Weighted MRI for Detection of Locally Recurrent Pancreatic Cancer After Surgical Resection. AJR Am J Roentgenol 2022; 219:762-773.
2. Lee S, Kim SH, Park HK, Jang KT, Hwang JA, Kim S. Pancreatic Ductal Adenocarcinoma: Rim Enhancement at MR Imaging Predicts Prognosis after Curative Resection. Radiology 2018; 288:456-466.
3. Yoon SH, Lee JM, Cho JY, et al. Small (≤ 20 mm) pancreatic adenocarcinomas: analysis of enhancement patterns and secondary signs with multiphasic multidetector CT. Radiology 2011; 259:442-452.
4. Ruiz A, Lemoinne S, Carrat F, Corpechot C, Chazouillères O, Arrivé L. Radiologic course of primary sclerosing cholangitis: assessment by three-dimensional magnetic resonance cholangiography and predictive features of progression. Hepatology 2014; 59:242-250.
5. Bai X, Wu L, Dai J, et al. Rim Enhancement and Peripancreatic Fat Stranding in Preoperative MDCT as Predictors for Occult Metastasis in PDAC Patients. Acad Radiol 2023; 30:2954-2961.
6. Kim H, Kim DH, Song IH, et al. Identification of intratumoral fluid–containing area by magnetic resonance imaging to predict prognosis in patients with pancreatic ductal adenocarcinoma after curative resection. Eur Radiol 2022; 32:2518-2528.
